# Associations of body composition parameters with postoperative outcome and perineural tumour invasion after oncological pancreatic resection

**DOI:** 10.1186/s12893-024-02457-5

**Published:** 2024-06-04

**Authors:** Tara C. Mueller, Martin Henselmann, Stefan Reischl, Carsten Jaeger, Charlotte Trefzer, Ihsan Ekin Demir, Helmut Friess, Marc E. Martignoni

**Affiliations:** 1 Department of Surgery, Klinikum rechts der Isar, TUM School of Medicine and Health, Technical University of Munich, Ismaninger Strasse 22, 81675 Munich, Germany; 2 Institute of Diagnostic and Interventional Radiology, Klinikum rechts der Isar, TUM School of Medicine and Health, Technical University of Munich, Ismaninger Strasse 22, 81675 Munich, Germany

**Keywords:** Pancreatic cancer, Body composition, Perineural tumour invasion, Postoperative complications, Malnutrition

## Abstract

**Background:**

Pancreatic cancer is often accompanied by wasting conditions. While surgery is the primary curative approach, it poses a substantial risk of postoperative complications, hindering subsequent treatments. Therefore, identifying patients at high risk for complications and optimizing their perioperative general condition is crucial. Sarcopenia and other body composition abnormalities have shown to adversely affect surgical and oncological outcomes in various cancer patients. As most pancreatic tumours are located close to the neuronal control centre for the digestive tract, it is possible that neural infiltration in this area deranges bowel functions and contributes to malabsorption and malnutrition and ultimately worsen sarcopenia and weight loss.

**Methods:**

A retrospective analysis of CT scans was performed for pancreatic cancer patients who underwent surgical tumour resection at a single high-volume centre from 2007 to 2023. Sarcopenia prevalence was assessed by skeletal muscle index (SMI), and visceral obesity was determined by the visceral adipose tissue area (VAT). Obesity and malnutrition were determined by the GLIM criteria. Sarcopenic obesity was defined as simultaneous sarcopenia and obesity. Postoperative complications, mortality and perineural tumour invasion, were compared among patients with body composition abnormalities.

**Results:**

Of 437 patients studied, 46% were female, the median age was 69 (61;74) years. CT analysis revealed 54.9% of patients with sarcopenia, 23.7% with sarcopenic obesity and 45.9% with visceral obesity. Sarcopenia and sarcopenic obesity were more prevalent in elderly and male patients. Postoperative surgical complications occurred in 67.7% of patients, most of which were mild (41.6%). Severe complications occurred in 22.7% of cases and the mortality rate was 3.4%. Severe postoperative complications were significantly more common in patients with sarcopenia or sarcopenic obesity. Visceral obesity or malnutrition based on BMI alone, did not significantly impact complications. Perineural invasion was found in 80.1% of patients and was unrelated to malnutrition or body composition parameters.

**Conclusions:**

This is the first and largest study evaluating the associations of CT-based body mass analysis with surgical outcome and histopathological perineural tumour invasion in pancreatic cancer patients. The results suggest that elderly and male patients are at high risk for sarcopenia and should be routinely evaluated by CT before undergoing pancreatic surgery, irrespective of their BMI. Confirmation of the results in prospective studies is needed to assess if pancreatic cancer patients with radiographic sarcopenia benefit from preoperative amelioration of muscle mass and function by exercise and nutritional interventions.

**Supplementary Information:**

The online version contains supplementary material available at 10.1186/s12893-024-02457-5.

## Introduction

### Background/rationale

Pancreatic cancer is one of the deadliest cancer entities and is frequently associated with tumour cachexia, sarcopenia, and nutritional issues due to its anatomical location and strong accompanying inflammatory and metabolic reactions. Margin-free (R0) surgical resection is still the only curative treatment option but is possible in only 10–20% of patients due to late diagnosis [1, 2]. Oncological resection is a major surgical procedure (pancreatoduodenectomy, distal pancreatectomy or total pancreatectomy) associated with correspondingly high postoperative morbidity and mortality rates. Postoperative complications occur in 50–80% of patients, and severe complications occur in 15–20% of patients [3].

#### Postoperative complications

The most frequent surgical complications after oncological pancreatic resection include the development of a clinically relevant pancreatic fistula [4] (CR-POPF, defined as grade B or C according to the International Society for Global Oncology [ISGPS] Definition 2016) [5], postoperative hemorrhage, surgical site infections (SSI) and mechanical problems such as bowel obstruction. CR-POPFs occur in up to 27% of distal pancreatectomies [3]. In addition, patients frequently suffer from cardiopulmonary complications because the surgeries are often long, and the preoperative performance status of the patients is often low. Not only can those complications be life-threatening; if they occur, they impede adjuvant therapy and have recently been shown to reduce disease-free survival [6]. The severity of surgical complications is commonly classified according to Clavien‒Dindo (CD 1–5): complications CD 1 and 2 are considered mild; complications CD 3a, 3b, 4a, and 4b are considered severe/life-threatening; and CD 5 means death of the patient [7].

#### Body composition

Computed tomography (CT) scans permit the separation of specific body mass compartments, such as skeletal muscle mass (SM), subcutaneous adipose tissue (SAT), visceral adipose tissue (VAT) and intermuscular adipose tissue mass (IMAT) compartments. For the measurement of SM, CT assessment is considered the gold standard [8, 9]. Pancreatic cancer patients routinely undergo abdominal CT scans, so their body composition changes can be evaluated over time and in correlation with disease progression and treatment response without any additional examinations or radiation exposure. Many pancreatic cancer patients are obese (BMI ≥ 30 kg/m^²^) at first presentation, so preoperative sarcopenia can be hidden (sarcopenic obesity) [8, 10]. A high amount of VAT (visceral obesity) is associated with a threefold-fold greater risk of developing cardiovascular disease (CVD) and a fivefold-fold greater risk of developing diabetes [11], as the tissue is metabolically active. Insulin resistance, metabolic syndrome, and increased proinflammatory factor levels are associated with carcinogenesis. Therefore, VAT should be analysed in addition to SM to evaluate the overall health and nutritional status of patients [11, 12].

Multiple recent studies have shown the negative influence of a preoperative low SM, sarcopenic obesity and visceral obesity on the postoperative course and overall survival of abdominal cancer patients [13–19]. A meta-analysis from 2019 included 70 international studies with 21,875 patients who evaluated the effect of CT-based diagnosis of sarcopenia on the short- and long-term outcomes of abdominal surgical patients. This analysis revealed associations of sarcopenia with overall survival, disease-free survival, total complications and major complications [20]. Another systematic review and meta-analysis confirmed the impact of low SM on oncological outcomes in pancreatic cancer patients, whereas the impact of SM on surgical outcomes remains to be established [21]. Recently, another systematic review focused on studies investigating the relationship of sarcopenia (CT-based diagnosis) with the incidence of CR-POPFs after pancreatic surgery. Twenty-one studies published between 2016 and 2021 with a total of 4,068 patients were identified. The results showed no clear differences in the incidence of CR-POPFs between sarcopenic and non-sarcopenic patients [4]. In contrast, a more recent retrospective analysis of 129 patients (30% of whom had pancreatic cancer) who underwent pancreaticoduodenectomy showed that preoperative, CT-based diagnosis of sarcopenia and visceral obesity were significant predictors of CR-POPFs and a high comprehensive complication score (CCI) [22]. These inconclusive results might be attributed to the small sample sizes, heterogeneous study populations and indications, and above all, the non-standardized measurement parameters and cut-off values used for sarcopenia and other body composition abnormalities determined by CT [4, 10].

#### Perineural tumour invasion

Another parameter that is associated with aggressive tumour biology, pain and poorer survival is perineural tumour invasion (Pn-Status), which is a characteristic histopathological feature of ductal pancreatic adenocarcinoma [23–25]. Prevalence rates (Pn1) in pancreatic cancer patient studies range from 45 to 100% [25]. In 2014, Liebl et al. proposed going one step further and evaluated not only the presence but also the severity of perineural invasion as a prognostic factor. The authors evaluated the “Neuronal Invasion (NI) Severity Score” in more than 2,000 patients with gastrointestinal tumours (132 patients with pancreatic cancer). Among those patients, those with pancreatic cancer had the highest NI Severity Score, which was confirmed to be an independent prognostic factor [25]. However, to our knowledge, whether there is a correlation between the incidence or severity of perineural invasion and sarcopenia or cancer cachexia has not yet been investigated. As the anatomical location of most pancreatic tumours is close to the *plexus coeliacus*, which is the neuronal control center for the digestive tract, it is possible that neural infiltration in this area can derange bowel functions and contribute to malabsorption and malnutrition and ultimately worsen sarcopenia and weight loss. Furthermore, this information could add to the identification of high-risk patients after surgery (as soon as complete histology results are available), which could benefit from increased postoperative monitoring, additional nutritional support and targeted therapies.

### Objective

To further improve perioperative risk evaluation and identify patients that would benefit from a multimodal treatment approach to improve their general/nutritional status prior to surgery, it is important to provide data from larger populations and develop standardized diagnostic algorithms. The objective of this study was to evaluate the relationship between preoperative sarcopenia, sarcopenic obesity, visceral obesity, malnutrition and postoperative complications in a large cohort of pancreatic cancer patients. Second, we explored the potential association of body composition abnormalities with perineural tumour invasion.

## Methods

### Study design, setting and participants

For this retrospective analysis, the prospective pancreatic cancer database of our institution was searched between July 2007 and February 2023. Patients were eligible for this study if they underwent curatively intended surgery and had undergone a routine diagnostic abdominal contrast-enhanced CT scan in venous phase (70 s after IV contrast application) that was taken a maximum of 45 days prior to surgery. The CT scan had to include the level of lumbar vertebra L3, and sufficient image quality was needed for the measurements. Furthermore, data on the postoperative course until hospital discharge, as well as the histological data, had to be available. No other eligibility criteria were applied. The manuscript was written in accordance with the STROBE Statement and Checklist [26].

### Variables and measurements

#### Demographic and surgical data

The following descriptive data were obtained for all patients: age (years), sex (F, M), American Society of Anesthesiologists (ASA) status (1–4), diabetes status (y/n), BMI (kg/m^2^), and malnutrition or obesity status (y/n). Furthermore, preoperative biliary stenting (y/n), type (pancreaticoduodenectomy, distal pancreatectomy or total pancreatectomy), duration of surgery (min), and type of neoadjuvant treatment (chemotherapy, radiotherapy, combination) were recorded. If postoperative surgical complications occurred within the hospital stay, their type and severity (Clavien-Dindo 1–5) [27] were documented. Furthermore, the incidence of CR-POPF (grade B/C) [5], postoperative biliary fistula, postoperative hemorrhage, delayed gastric emptying (DGE), surgical site infections (SSI) and relaparotomy were documented, as was the incidence of non-surgical complications and the length of postoperative hospital stay (days).

#### Definition of malnutrition and obesity

According to the Global Leadership Initiative on Malnutrition (GLIM), malnutrition is defined based on three phenotypic criteria (non-volitional weight loss, low BMI, and reduced SM) and two etiologic criteria (reduced food intake or assimilation and inflammation or disease burden). To diagnose malnutrition, at least one phenotypic criterion and one etiologic criterion should be met [28]. In this study, we defined “cancer” as the etiologic criterion, and the phenotypic criterion was “low BMI”. If both criteria were fulfilled, the subjects were classified as “malnourished”. According to the GLIM definition, “low BMI” was defined as < 20 kg/m^2^ if < 70 years or < 22 kg/m^2^ if ≥ 70 years, and obesity was defined as a BMI ≥ 30 kg/m^2^ [28].

#### Analysis of body composition by CT

CT analysis was performed on routinely obtained abdominal CT scans using the Slice-O-Matic® software V 5.0 (Tomovision, Montreal, Canada). The CT Hounsfield unit (HU) thresholds for different tissue types were used as follows: SM -29 to + 150 HU, IMAT and SAT -190 to -30 HU and VAT -150 to -50 HU. The total abdominal muscle area at the level of lumbar vertebra 3 (L3) was calculated and standardized for body height, resulting in the SM-Index (cm^2^/m^2^), which correlates with whole-body muscle mass as previously described [29–31]. The cut-off values for “low SMI” are still not standardized, but the most commonly used values are < 52.4 (cm^2^/m^2^) for men and < 38.5 (cm^2^/m^2^) for women, as defined by Prado et al. [13, 29]. Sarcopenic obesity was defined as “low SMI” and simultaneous BMI ≥ 30 kg/m^2^.

Similarly, the total area of VAT (cm^2^) at the L3 level can be divided by body height squared to obtain the VAT Index (VATI). The cut-off values for “visceral obesity” are even less standardized than for the SMI and vary widely in the literature [11, 12]. We used the following cut-off values: VAT > 163.8 cm^2^ for males and > 80.1 cm^2^ for females [32] as they were established for a cohort of patients similar to ours.

#### Histological parameters

The cancer type (adenocarcinoma, other), histological tumour stage (American Joint Committee on Cancer [AJCC] staging and TNM classification 8th edition, 2017) [33] and incidence of neural tumour invasion (Pn1) were recorded. The severity of perineural invasion was defined by the NI Severity Score and was previously analysed in 103 patients within a larger histopathological study [25].

### Statistical analyses

The statistical analysis and linear correlation of the data were performed with SPSS software V24.0 (IBM, USA). Quantitative variables are expressed as medians and interquartile ranges (P25; P75) due to asymmetric distributions. Qualitative variables are expressed as actual and relative frequencies. Differences between quantitative variables were assessed using the Mann‒Whitney U test. Qualitative variables were compared using Fisher’s exact test. Correlations were analysed using Spearman’s ρ. All analyses were performed with an explorative significance level of 5%. No adjustment for multiple comparisons was performed.

## Results

### Participants/study size

Between July 2007 and February 2023, 437 patients underwent curative surgery for pancreatic cancer at a single high-volume centre and met all the inclusion criteria. Another 200 patients were excluded due to insufficient quality of CT Scan and/or missing follow-up data (*n* = 144), or missing informed consent (*n* = 56) for the data collection and analysis.

### Descriptive data

Table 1 shows the descriptive data for all patients and compares those with and without radiographic (CT) sarcopenia. A total of 46% of the patients were female, and the median age was 69 (61; 74) years. The preoperative median ASA score was 2 (2; 3). The median BMI was 24.24 (22.22; 26.73). According to the GLIM criteria, 79 patients (18.1%) were malnourished, and 36 patients (8.2%) were obese at first presentation. In addition, 27.9% of all patients presented with diabetes. According to the applied cut-off values, analysis of the preoperative CT scans revealed that 54.9% of all patients were sarcopenic, 46.0% had visceral obesity and 23.8% presented with sarcopenic obesity. The median values for SMI, VAT and VATI were 44.48 (38.7; 50.73) cm^2^/m^2^, 113.20 (62.50; 182.43) cm^2^ and 39.07 (21.2; 62.07) cm^2^/m^2,^ respectively.

**Table 1:** Baseline data of all patients and patients with or without radiographic (CT) sarcopenia

		Total Patients (*n* = 437)	Sarcopenia (*n* = 240)	No sarcopenia (*n* = 197)	*p* value
Preoperative Status					
	Age (years) (median, LQ; UQ)	69 (61;74)	70 (63;75)	67 (60;73)	**0.014** ^**#**^
	Female (n, %)	201 (46%)	84 (35%)	117 (59.4%)	< 0.001*
	ASA Status (median, LQ; UQ)	2 (2;3)	2 (2;3)	2 (2;3)	0.147^#^
	BMI (kg/m^2^) (median, LQ; UQ)	24.2(22.2;26.7)	23.5 (21.2;25.4)	25.4(23.1;28.1)	**< 0.001** ^**#**^
	GLIM Malnutrition (n, %)	79 (18.1%)	61 (25.4%)	18 (9.1%)	< 0.001*
	Obesity (n, %)	36 (8.2%)	12 (5%)	24 (12.2%)	0.008*
	Diabetes (n, %)	122 (27.9%)	66 (27.5%)	56 (28.4%)	0.831*
	CT SMI (cm^2^/m^2^) (median, LQ; UQ)	44.5 (38.7;50.7)	41.9 (36.8;47.5)	47.1(41.3;55.8)	**< 0.001** ^**#**^
	CT VATI (cm^2^/m^2^) (median, LQ; UQ)	39.1(21.2;62.1)	38.9(19.8;59.2)	40.5(22.6;65.1)	0.502^#^
	CT VAT (cm^2^) (median, LQ; UQ)	113.2 (62.5;182.4)	113.8 (62.1;183.4)	113.2 (62.6;178.8)	0.888^#^
	Visceral obesity (n, %)	201 (46%)	104 (43.3%)	97 (49.2%)	0.247*
	Neoadjuvant treatment				
	Chemotherapy (n, %)	79 (18.1%)	41 (17.1%)	38 (19.3%)	0.618*
	Radiotherapy (n, %)	4 (0.9%)	1 (0.4%)	3 (1.5%)	0.332*
	Combination (n, %)	1 (0.2%)	0	1 (0.5%)	0.451*
	Preoperative biliary stenting (n, %)	145 (33.2%)	98 (40.8%)	47 (23.9%)	< 0.001*
**Operation**					
	Pancreaticoduodenectomy (n, %)	268 (61.3%)	161 (67.1%)	107 (54.3%)	0.008*
	Distal Pancreatectomy (n, %)	104 (23.8%)	45 (18.8%)	59 (29.9%)	0.007*
	Total Pancreatectomy (n, %)	65 (14.9%)	34 (14.2%)	31 (15.7%)	0.686*
	Duration of Operation (min) (median, LQ; UQ)	379 (302;451.5)	383 (317.8;455.5)	373 (284.5;450)	0.173^**#**^
**Histopathological data**					
	PDAC (n, %)	422 (96.6%)	235 (97.9%)	187 (94.9%)	0.114*
	Other (n, %)	15 (3.4%)	5 (2.1%)	10 (5.1%)	
	AJCC stage (8th edition)				0.975*
	0 (n, %)	3 (0.7%)	1 (0.4%)	2 (1.0%)	
	IA (n, %)	32 (7.3%)	18 (7.5%)	14 (7.1%)	
	IB (n, %)	62 (14.2%)	36 (15%)	26 (13.2%)	
	IIA (n, %)	22 (5%)	12 (5%)	10 (5.1%)	
	IIB (n, %)	140 (32%)	79 (32.9%)	61 (31%)	
	III (n, %)	144 (33%)	76 (31.7%)	68 (34.5%)	
	IV (n, %)	34 (7.8%)	18 (7.5%)	16 (8.1%)	
	Perineural tumour invasion				0.118*
	Pn0 (n, %)	87 (19.9%)	41 (17.1%)	46 (23.4%)	
	Pn1 (n, %)	350 (80.1%)	199 (82.9%)	151 (76.6%)	

Symbols: ^#^ Man-Whitney-U test; *Fisher’s exact test

*Abbreviations* ASA = American Society of Anesthesiologists; BMI = Body mass index; GLIM = Global leadership initiative on malnutrition; CT = Computed tomography; SMI = Skeletal muscle index, VAT = Visceral adipose tissue; VATI = Visceral adipose tissue index; PDAC = Pancreatic ductal adenocarcinoma; AJCC = American Joint Committee on Cancer

Furthermore, 19.2% of all patients received neoadjuvant therapy (79 chemotherapy, 4 radiotherapy, 1 combination). In addition, 33.2% of patients received preoperative biliary stenting. Pancreaticoduodenectomy was performed in 61.3% of all patients; 23.8% of the procedures involved distal pancreatectomies, and 14.9% involved total pancreatectomies. The median duration of operation was 379 (302; 451.5) minutes. The majority of patients were histologically diagnosed with pancreatic ductal adenocarcinoma (PDAC) (96.6%), 80.1% of whom exhibited perineural invasion (Pn1). A total of 3.4% had other histological subtypes of pancreatic cancer, such as anaplastic carcinoma, neuroendocrine carcinoma, micropapillary tumour, cystadenocarcinoma, adenosquamous carcinoma or acinar cell carcinoma. According to the AJCC staging system, most patients had stage III tumours (33%). Milder stages, such as 0, IA, and IB, were documented in only 0.7%, 7.3% and 14.2%, respectively. Stage IIA accounted for 5.0%, and stage IIB accounted for 32.0%. A total of 7.8% of patients were classified as stage IV postoperatively.

As shown in Table 1, the following differences between the patients with and without radiographic sarcopenia were statistically significant: Patients with sarcopenia were older (*p* = 0.014), more likely to be male (*p* < 0.001) and had a lower mean BMI (*p* < 0.001); thus, they had a greater incidence of GLIM malnutrition (*p* < 0.001) while being less frequently obese (*p* = 0.008). Preoperative biliary stenting was significantly more frequent in the group with sarcopenia (40.8% vs. 23.9%, *p* < 0.001). And a significantly greater number of patients with sarcopenia underwent pancreaticoduodenectomy (*p* = 0.008), while fewer patients underwent distal pancreatectomy (*p* = 0.007).

The same descriptive variables were compared for patients with radiographic visceral obesity and sarcopenic obesity, as well as GLIM malnutrition; the full corresponding tables can be found in Supplementary Materials 1–3. The results are summarized as follows.

Patients with visceral obesity were significantly older (*p* < 0.001), had a greater mean BMI (*p* < 0.001), were more likely to be obese (*p* < 0.001), and had a greater incidence of diabetes (*p* < 0.001) than patients without visceral obesity. As the malnutrition criteria were based on BMI, patients were less frequently classified as malnourished (*p* < 0.001). SMI values were not significantly different between patients with or without visceral obesity. Moreover, regarding the surgical and histopathological data, there were no significant differences between patients with or without visceral obesity.

The patients in the sarcopenic obesity group were significantly older (*p* < 0.001), more likely to be male (*p* < 0.001) and more likely to have a higher average preoperative ASA score (*p* = 0.001) than patients without sarcopenic obesity. Furthermore, a greater proportion of patients in this group had diabetes (*p* = 0.033). VAT values were greater in this group than in the control group without sarcopenic obesity (*p* < 0.001). Moreover, significantly fewer patients with sarcopenic obesity had undergone chemotherapy within the first three months before surgery (*p* = 0.028).

The patients classified as “malnourished” according to the GLIM criteria (*n* = 79) were significantly older (*p* = 0.006) and included more women (*p* < 0.001). The SMI, VATI and VAT values were all significantly lower in patients with malnutrition defined by low BMI (*p* < 0.001). Significant differences were also observed regarding the AJCC stage (*p* = 0.016), which was greater in patients with malnutrition. Moreover, no differences in the baseline data between patients with and without GLIM malnutrition were found.

### Outcome data

As shown in Table 2, postoperative surgical complications occurred in 67.7% of the patients. Most of the patients had mild complications (41.6% CD 1–2), 22.7% had severe complications (CD 3–4), and 3.4% died during the postoperative course (CD 5). Patients with sarcopenia had significantly more severe complications according to the one-sided t test (25.8% vs. 18.8%; *p* = 0.05). Moreover, there were no statistically significant differences between the two groups.

**Table 2:** Incidence and severity of postoperative complications in patients with or without radiographic (CT) sarcopenia

	Total patients (*n* = 437)		Sarcopenia (*n* = 240)		No sarcopenia (*n* = 197)		*p* value
	n	%	n	%	n	%	
**Surgical complications**	296	67.7	167	69.6	129	65.5	0.411**
* Mild (CD 1–2)*	182	41.6	94	39.2	88	44.7	0.283**
* Severe (CD 3–4)*	99	22.7	62	25.8	37	18.8	0.05*
* Death (CD 5)*	15	3.4	11	4.6	4	2	0.189**
CR-POPF (B/C)	37	8.5	23	9.6	14	7.1	0.392**
Postoperative biliary fistula	12	2.7	9	3.8	3	1.5	0.239**
Postoperative hemorrhage	32	7.3	18	7.5	14	7.1	1.00**
Surgical Site Infection	55	12.6	33	13.8	22	11.2	0.47**
* Incisional (superficial, deep)*	23	5.3	15	6.2	8	4.1	0.391**
* Organ/Space*	32	7.3	18	7.5	14	7.1	1.00**
DGE	53	12.1	31	12.9	22	11.2	0.659**
Reoperation	32	7.3	19	7.9	13	6.6	0.713**
**Other complications**	153	35	82	34.2	71	36	0.688**
Diarrhea	57	13	24	10	33	16.8	0.045**
Vomiting	14	3.2	2	0.8	12	6.1	0.002**
**Postoperative hospital stay** (median, LQ; UQ) (days)	17 (12;23)		17 (13;24)		16 (11.5;22)		0.058^#^

Symbols: ^#^ Man-Whitney-U test; *Fisher’s exact test (one-sided); **Fisher’s exact test (two-sided)

*Abbreviations* CD = Clavien.Dindo; CR-POPF = Clinically relevant postoperative pancreatic fistula; DGE = delayed gastric emptying

CR-POPFs were documented in 8.5% of all patients, and postoperative biliary fistulas were documented in 2.7%. Postoperative hemorrhage occurred in 7.3%, SSI (I-III) occurred in 12.6%, 12.1% of patients had postoperative DGE, and 7.3% required relaparotomy. The median length of postoperative hospital stay was 17 (12;23) days. Regarding these complications and the length of hospital stay, there were no significant differences between patients with or without sarcopenia. Furthermore, 35% of all patients suffered from relevant non-surgical complications, such as gastrointestinal symptoms (16.2%), ascites (8.0%), cardiovascular complications (3.7%), pulmonary events (3.4%), or acute renal or urinary problems (6.9%). There were no significant differences between patients with or without sarcopenia, except for vomiting and diarrhoea, which were both more common in patients without sarcopenia.

The same outcome variables were compared for patients with visceral obesity, sarcopenic obesity and GLIM malnutrition vs. patients without those features. The corresponding tables are contained in Supplementary Materials 4–6. For patients with visceral obesity, no significant differences were found regarding postoperative surgical complications, mortality rate or length of hospital stay. Regarding non-surgical postoperative complications, significantly more patients with visceral obesity experienced renal complications (*p* = 0.015) and pulmonary embolism (*p* = 0.004).

Patients with sarcopenic obesity had a significantly greater mortality rate (6.7% vs. 2.4%) and a greater incidence of SSIs (18.3% vs. 10.8%) than patients without sarcopenic obesity. These expected differences were statistically significant according to a one-sided t-test (*p* = 0.042 and *p* = 0.037, respectively). Furthermore, postoperative biliary fistula was more frequent in patients with sarcopenic obesity (*p* = 0.01), while there was no significant difference in the incidence of CR-POPFs. In addition, there was an increased incidence of DGE (*p* = 0.038), postoperative pneumonia (*p* = 0.021), and postoperative pulmonary embolism (*p* = 0.01) in patients with sarcopenic obesity. These patients also had longer postoperative hospital stays, with a median of 18.5 (14; 28.75) days *versus* 16 (12; 21) days, in patients without sarcopenic obesity (*p* < 0.001). Finally, patients without sarcopenic obesity experienced significantly more postoperative diarrhoea (*p* = 0.03).

There were no significant differences in postoperative surgical complications between patients with GLIM malnutrition and patients with a higher BMI (no GLIM malnutrition), except for a greater incidence of postoperative biliary fistulas (*p* = 0.047). The mortality rate, length of hospital stay, and incidence of non-surgical postoperative complications were not significantly different between patients with and without GLIM malnutrition.

### Subgroup analysis

According to the subgroup analysis of patients with available data on the histopathological NI Severity Score (*n* = 103), there was no significant correlation between the NI Severity Score and the SMI (*p* = 0.568) or VATI (*p* = 0.611) from the CT scans. The correlation coefficient was positive in both tests (,057) and (,051). As shown in Table 3, when patients with malnutrition, visceral obesity, sarcopenic obesity or sarcopenia were compared to patients without these features, there was no significant difference in the NI Severity Score. To additionally investigate the potential impact of perineural invasion on bowel motility and function, the status (Pn1 vs. Pn0) and NI Severity Score were correlated with clinical gastrointestinal symptoms (DGE, diarrhea and vomiting). This analysis showed no statistically significant associations between these factors either. The corresponding table can be found in Supplemental Material 7.

**Table 3:** Subgroup analysis of the severity of perineural tumour invasion (NI severity score) and body composition parameters

		NI Severity Score		
		*n* = 103	Median (LQ; UQ)	*p* value
**Sarcopenia**	*Yes*	54	8 (4.23;14.78)	0.724^#^
	*No*	49	9.3 (4;15.85)	
**Visceral obesity**	*Yes*	41	9 (4.15;14.65)	1.000^#^
	*No*	62	8.5 (4;15.4)	
**Sarcopenic obesity**	*Yes*	22	6 (2.53;10.55)	0.070^#^
	*No*	81	9.7 (4.85;15.7)	
**GLIM malnutrition**	*Yes*	24	9.3 (4.25;19.53)	0.292^#^
	*No*	79	8.7 (4;14)	

Symbols: ^#^ Man-Whitney-U test

*Abbreviations* NI = neural invasion; GLIM = Global leadership initiative on malnutrition

## Discussion

### Key results

To our knowledge, this is the first and largest cohort of surgically resectable pancreatic cancer patients in which CT-based abnormalities in body composition and their associations with postoperative complications and histopathological data were evaluated. The key results of this retrospective analysis showed that, while no association of histopathological data was observed, body composition parameters were associated with specific clinical features and surgical outcomes. While sarcopenia, especially in combination with obesity, was a risk factor for higher postoperative morbidity and mortality, visceral obesity was associated with higher rates of general complications (e.g., PE and renal complications). All body composition abnormalities were more frequent in older and male patients. However, malnutrition defined by BMI alone was more common in female patients than in male patients and did not negatively impact surgical outcomes.

### Limitations

The limitations of the present study include its retrospective and monocentric study design. No data on muscle function were available because of this. Functional tests of muscle strength and physical performance status would add to the diagnostic accuracy of sarcopenia, as CT scans do not include peripheral muscle mass stores. Furthermore, the optimal cut-off values for CT-based sarcopenia diagnosis have yet to be established, as body composition varies between sex and ethnicity (e.g., values from Asian cohorts cannot simply be transferred to Caucasians). This well-known problem has been addressed in other similar studies. We decided to use the sex-specific cut-off values determined by optimal stratification by Prado et al., as these values are the most frequently used values in comparable European-based studies [12, 20]. Interestingly, the only other study that investigated body composition in a homogenous cohort of pancreatic head cancer patients (*n* = 199) similar to the present study, established their own cut-off values by tertiles, and the values were significantly different from those previously published (45.1 cm^2^/m^2^ for males and 36.9 cm^2^/m^2^ for females) [19]. The same problem applies to the diagnosis of visceral obesity by CT, which is even more confusing, as some studies use VAT and others use VATI. Many previous studies have used a cut-off value > 100cm^2^ for VAT from the Japanese Society for the Study of Obesity [12]. However, this value applies to Asian populations, only. A recent publication by Baggermann and colleagues determined cut-off values for visceral obesity in a cohort of Caucasian healthy kidney donors and showed that a VATI > 38.7 cm^2^/m^2^ for males and 24.9 cm^2^/m^2^ was associated with increased metabolic risk, with a sensitivity of 80% [11]. We decided to use the cut-off values established by Doyle et al., as the population of gastrointestinal cancer patients was more comparable to our cohort [32]. The abovementioned study of Dutch pancreatic cancer patients determined much higher cut-off values of VATI > 68.2 (cm^2^/m^2^) for males and 39.2 (cm^2^/m^2^) for females [19]. Regarding the secondary analysis of perineural tumour invasion, a limitation was that the NI Severity Score was available for a small subset of patients, only.

### Interpretation

The results of this analysis are compatible with the majority of previously conducted studies regarding preoperative sarcopenia, as shown by a recent large meta-analysis of 70 studies including 21,875 surgical patients (with benign and malignant diagnoses). The overall median incidence of sarcopenia in those studies was 34.7% (range from 2.1 to 83.3%), although as many as 19 different cut-off values were used for the definition. Preoperative sarcopenia was associated with an increased risk of postoperative complications and mortality [20]. A systematic review that included patients with abdominal malignancies only, revealed that clinical outcomes (survival time, postoperative complications, systemic inflammation) were significantly and adversely influenced by sarcopenia (measured by CT) in 7 of the 10 studies included. However, different cut-off values were used, and most of the included studies did not include functional muscle testing [13]. Another recent retrospective, multicenter analysis compared CT body composition parameters before and after neoadjuvant therapy with the surgical outcome of pancreatic cancer patients (*n* = 121). The authors found that patients who achieved a SMI gain from 35 to 40 cm^2^/m^2^ during therapy had up to 60% lower odds of postoperative complications. Patients who already had an SMI greater than 40 cm^2^/m^2^ at first presentation did not benefit from further gain [34]. These results confirm the importance of identifying high-risk patients with sarcopenia or sarcopenic obesity as early as possible to allow them to benefit from individual preoperative exercise and nutritional interventions. As every pancreatic cancer patient receives an abdominal staging CT, analysis of body composition could be easily effectuated at almost no additional costs. Technical advances in artificial intelligence (AI) have led to excellent speed and accuracy in analysing body composition on CT scans, and several fully automated CT-based body composition analyses have already been developed [12].

A similar meta-analysis of visceral obesity was performed, in which 19 studies with 3,528 patients were included. Of those, 46% had visceral obesity. However, 12 of the 19 studies used the “Asian” cut-off value of VAT > 100 cm^2^, irrespective of sex or ethnicity. There were no significant differences between patients with visceral obesity and those with normal VAT for 30-day mortality or overall postoperative complications. In contrast to our results, however, this analysis did demonstrate an association between visceral obesity and increased incidence of SSI, pneumonia, and POPF [35].

A position paper of the International Study Group on Pancreatic Surgery (ISGPS) from 2018 recommended that the nutritional status should be part of routine preoperative assessment and should include not only BMI and weight loss but also the measurement of sarcopenia, which could otherwise be hidden in obese patients [4, 36]. Furthermore, reports have shown that chemotherapy regimens based on body composition are superior to those based on BMI alone for reducing toxicity [10]. Our findings confirm this recommendation, as when we compared patients by BMI only, no significant differences in surgical outcomes were detected. According to a review by Gibson et al., the number of patients diagnosed with sarcopenia using CT was 27.3–66.7% greater than the number of patients identified as malnourished using BMI only [13]. However, even if BMI and weight loss alone are not sufficient for preoperative risk assessment but they may still be useful indicators of underlying sarcopenia. 

## Conclusion

To our knowledge, this is the first and largest study evaluating the association of postoperative complications with CT-based body mass analysis and malnutrition, as well as histopathological perineural tumour invasion, in a homogeneous cohort of surgical pancreatic cancer patients. The results of this retrospective analysis suggest that especially elderly, male and obese patients should be routinely evaluated for sarcopenia before undergoing pancreatic surgery, independent of their BMI. The role of visceral obesity as pre-operative risk factor remains to be established.

The results of the smaller subgroup analysis on perineural tumour invasion showed no association with body composition abnormalities or clinical gastrointestinal symptoms.

Although confirmation of our data in prospective studies is needed, patients with manifest sarcopenia would certainly benefit from preoperative amelioration of muscle mass and function by exercise and nutritional interventions. Therefore, screening for sarcopenia via preoperative CT should be integrated into routine preparation for pancreatic surgery. In the future, this could be realized by using artificial intelligence (AI) approaches, which will ultimately facilitate the establishment of more accurate population-based reference values.

### Electronic supplementary material

Below is the link to the electronic supplementary material.

Supplementary Material 1

## Data Availability

All the data generated or analyzed during this study are included in this published article and its supplementary material.

## Abbreviations

95% CI: 95% confidence interval
BMI: Body mass index
VAT: Visceral adipose tissue area
VATI: Visceral adipose tissue index
SM: Skeletal muscle mass
SAT: Subcutaneous adipose tissue
IMAT: Intermuscular adipose tissue mass
CT: Computed tomography
GLIM: Global Leadership Initiative on Malnutrition
HU: Houndsfield unit
L3: Lumbar vertebra 3
CCI: Comprehensive complication index
Pn: Perineural invasion
NI: Neural invasion
STROBE: Strengthening the reporting of observational studies in epidemiology
ASA: American Society of Anesthesiologists
DGE: Delayed gastric emptying
SSI: Surgical site infection
AJCC: American joint committee on cancer
PDAC: Pancreatic ductal adenocarcinoma
LQ: Lower quartile
MM: Muscle mass
SMI: Skeletal muscle index
UQ: Upper quartile
CD: Clavien‒Dindo
CR-POPF: Clinically relevant postoperative pancreatic fistula
ISGPS: International study group for pancreatic surgery
PE: Pulmonary embolism
AI: Artificial intelligence
