# Supplementary material for: Associations of body composition parameters with postoperative outcome and perineural tumour invasion after oncological pancreatic resection
Source: BMC Surg. 2024 Jun 4;24:175. doi: 10.1186/s12893-024-02457-5 (PMC11149203; doi:10.1186/s12893-024-02457-5)
Supplement: Supplementary file 1 — Supplementary Material 1 [file 12893_2024_2457_MOESM1_ESM.docx]

**Supplemental Material to Manuscript:**

“**Associations of body composition parameters with postoperative outcome and perineural tumour invasion after oncological pancreatic resection”**

**CONTENTS**

**SUPPLEMENTAL TABLE 1:** **Baseline data of all patients, patients with or without radiographic (CT) visceral obesity**

**SUPPLEMENTAL TABLE 2: Baseline data of all patients, patients with or without radiographic (CT) sarcopenic obesity**

**SUPPLEMENTAL TABLE 3: Baseline data of all patients, patients with or without GLIM malnutrition**

**SUPPLEMENTAL TABLE 4: Incidence and severity of postoperative complications in patients with or without radiographic (CT) visceral obesity**

**SUPPLEMENTAL TABLE 5: Incidence and severity of postoperative complications in patients with or without radiographic (CT) sarcopenic obesity**

**SUPPLEMENTAL TABLE 6: Incidence and severity of postoperative complications in patients with or without GLIM malnutrition**

**SUPPLEMENTAL TABLE 7: Correlation of perineural tumour invasion and postoperative clinical gastrointestinal symptoms**

**SUPPLEMENTAL TABLE 1:**

**Baseline data of all patients. patients with or without radiographic (CT) visceral obesity**

|  | **Total Patients (n=437)** | **With visceral obesity (n=201)** | **Without visceral obesity (n=236)** | **P value** |
| --- | --- | --- | --- | --- |
| **Preoperative Status** | | | | |
| Age (years) (median, LQ;UQ) | 69 (61;74) | 70 (63.5;75.5) | 67 (59;73) | .000**^#^** |
| Female (n, %) | 201 (46.0%) | 93 (46.3%) | 108 (45.8%) | .924***** |
| ASA Status (median, LQ;UQ) | 2 (2;3) | 2 (2;3) | 2 (2;3) | .059***** |
| BMI (kg/m^2^) (median, LQ;UQ) | 24.24 (22.22;26.73) | 25.71 (24.14;28.40) | 22.89 (20.47;24.93) | .000**^#^** |
| GLIM Malnutrition (n, %) | 79 (18.1%) | 13 (6.5%) | 66 (28%) | .000***** |
| Obesity (n, %) | 36 (8.2%) | 33 (16.4%) | 3 (1.3%) | .000***** |
| Diabetes (n, %) | 122 (27.9%) | 73 (36.3%) | 49 (20.8%) | .000***** |
| CT SMI (cm^2^/m^2^)  (median, LQ;UQ) | 44.48 (38.70;50.73) | 44.71 (39.11;51.47) | 44.03 (38.05;50.03) | .221**^#^** |
| CT VATI (cm^2^/m^2^)  (median, LQ;UQ) | 39.07 (21.20;62.07) | 65.31 (47.01;81.96) | 23.49 (11.89;36.09) | .000**^#^** |
| CT VAT (cm^2^)  (median, LQ;UQ) | 113.20 (62.50;182.43) | 189.30 (124.63;238.13) | 63.76 (34.63;110.43) | .000**^#^** |
| Neoadjuvant treatment |  |  |  |  |
| Chemotherapy (n, %) | 79 (18.1%) | 31 (15.4%) | 48 (20.3%) | .213***** |
| Radiotherapy (n, %) | 4 (0.9%) | 3 (1.5%) | 1 (0.4%) | .338***** |
| Combination (n, %) | 1 (0.2%) | 0 | 1 (0.4%) | 1.000***** |
| Preoperative biliary stenting (n, %) | 145 (33.2%) | 69 (34.3%) | 76 (32.2%) | .684***** |
| **Operation** |  |  |  |  |
| Pancreaticoduodenectomy (n, %) | 268 (61.3%) | 121 (60.2%) | 147 (62.3%) | .694***** |
| Distal Pancreatectomy (n, %) | 104 (23.8%) | 50 (24.9%) | 54 (22.9%) | .653***** |
| Total Pancreatectomy (n, %) | 65 (14.9%) | 30 (14.9%) | 35 (14.8%) | 1.000***** |
| Duration of Operation (min)  (median, LQ;UQ) | 379 (302;451.5) | 382.00 (302.50;452.00) | 377.50 (302.00;451.75) | .749***** |
| **Histopathological data** |  |  |  |  |
| PDAC (n, %) | 422 (96.6%) | 195 (97.0%) | 227 (96.2%) | .794***** |
| Other (n, %) | 15 (3.4%) | 6 (3%) | 9 (3.8%) |  |
| AJCC stage (8^th^ edition) |  | | | |
| 0 (n, %) | 3 (0.7%) | 1 (0.5%) | 2 (0.8%) | .094***** |
| IA (n, %) | 32 (7.3%) | 19 (9.5%) | 13 (5.5%) |  |
| IB (n, %) | 62 (14.2%) | 23 (11.4%) | 39 (16.5%) |  |
| IIA (n, %) | 22 (5%) | 15 (7.5%) | 7 (3.0%) |  |
| IIB (n, %) | 140 (32.0%) | 63 (31.3%) | 77 (32.6%) |  |
| III (n, %) | 144 (33.0%) | 68 (33.8%) | 76 (32.2%) |  |
| IV (n, %) | 34 (7.8%) | 12 (6.0%) | 22 (9.3%) |  |
| Perineural tumour invasion |  |  |  |  |
| Pn0 (n, %) | 87 (19.9%) | 48 (23.9%) | 39 (16.5%) | .071***** |
| Pn1 (n, %) | 350 (80.1%) | 153 (76.1%) | 197 (83.5%) |  |
| Symbols: ^#^ Man-Whitney-U test; *Fisher’s exact test  Abbreviations: ASA = American Society of Anesthesiologists; BMI = Body mass index; GLIM = Global leadership initiative on malnutrition; CT = Computed tomography; SMI = Skeletal muscle index, VAT = Visceral adipose tissue; VATI = Visceral adipose tissue index; PDAC = Pancreatic ductal adenocarcinoma; AJCC = American Joint Committee on Cancer | | | | |

**SUPPLEMENTAL TABLE 2:**

**Baseline data of all patients, patients with or without radiographic (CT) sarcopenic obesity**

|  | **Total Patients (n=437)** | **With sarcopenic obesity (n=104)** | **Without sarcopenic obesity (n=333)** | **P value** |
| --- | --- | --- | --- | --- |
| **Preoperative Status** | | | | |
| Age (years) (median, LQ;UQ) | 69(61;74) | 72(64.25;76) | 68 (60;73.5) | .000***** |
| Female (n, %) | 201 (46.0%) | 32 (30.8%) | 169 (50.8%) | .000***** |
| ASA Status (median, LQ;UQ) | 2 (2;3) | 2 (2;3) | 2 (2;3) | .001**^#^** |
| BMI (kg/m^2^) (median, LQ;UQ) | 24.24 (22.22;26.73) | 24.83 (23.39;26.92) | 23.92 (21.64;26.64) | .003**^#^** |
| GLIM Malnutrition (n, %) | 79 (18.1%) | 10 (9.6%) | 69 (20.7%) | .009***** |
| Obesity (n, %) | 36 (8.2%) | 12 (11.5%) | 24 (7.2%) | .159***** |
| Diabetes (n, %) | 122 (27.9%) | 38 (36.5%) | 84 (25.2%) | .033***** |
| CT SMI (cm^2^/m^2^)  (median, LQ;UQ) | 44.48 (38.70;50.73) | 24.54 (37.05;48.57) | 44.64 (39.13;52.49) | .000**^#^** |
| CT VATI (cm^2^/m^2^)  (median, LQ;UQ) | 39.07 (21.20;62.07) | 65.31 (50.70;81.50) | 31.47 (16.62;48.01) | .000**^#^** |
| CT VAT (cm^2^)  (median, LQ;UQ) | 113.20 (62.50;182.43) | 197.93 (157.01;240.54) | 92.32 (47.94;142.25) | .000**^#^** |
| Visceral obesity (n, %) | 201 (46.0%) | 104 ((100%) | 97 (29.1%) | .000***** |
| Neoadjuvant treatment |  |  |  |  |
| Chemotherapy (n, %) | 79 (18.1%) | 11 (10.6%) | 68 (20.4%) | .028***** |
| Radiotherapy (n, %) | 4 (0.9%) | 0 | 4 (1.2%) | .577***** |
| Combination (n, %) | 1 (0.2%) | 0 | 1 (0.3%) | 1.00***** |
| Preoperative biliary stenting (n, %) | 145 (33.2%) | 42 (40.4%) | 103 (30.9%) | .095***** |
| **Operation** |  | | | |
| Pancreaticoduodenectomy (n, %) | 268 (61.3%) | 69 (66.3%) | 199 (59.8%) | .250***** |
| Distal Pancreatectomy (n, %) | 104 (23.8%) | 21 (20.2%) | 83 (24.9%) | .358***** |
| Total Pancreatectomy (n, %) | 65 (14.9%) | 14 (13.5%) | 51 (15.3%) | .753***** |
| Duration of Operation (min)  (median, LQ;UQ) | 379 (302;451.5) | 386.5(321.25;452) | 378(298.00;451.5) | .435**^#^** |
| **Histopathological data** |  | | | |
| PDAC (n, %) | 422 (96.6%) | 102 (98.1%) | 320 (96.1%) | .538***** |
| Other (n, %) | 15 (3.4%) | 2 (1.9%) | 13 (3.9%) |  |
| AJCC stage (8^th^ edition) |  | | | |
| 0 (n, %) | 3 (0.7%) | 0 (0.0%) | 3 (0.9%) | .754***** |
| IA (n, %) | 32 (7.3%) | 10 (9.6%) | 22 (6.6%) |  |
| IB (n, %) | 62 (14.2%) | 13 (12.5%) | 49 (14.7%) |  |
| IIA (n, %) | 22 (5%) | 7 (6.7%) | 15 (4.5%) |  |
| IIB (n, %) | 140 (32.0%) | 32 (30.8%) | 108 (32.4%) |  |
| III (n, %) | 144 (33.0%) | 36 (34.6%) | 108 (32.4%) |  |
| IV (n, %) | 34 (7.8%) | 6 (5.8%) | 28 (8.4%) |  |
| Perineural tumour invasion |  |  |  |  |
| Pn0 (n, %) | 87 (19.9%) | 17 (16.3%) | 70 (21.0%) | .328***** |
| Pn1 (n, %) | 350 (80.1%) | 87 (83.7%) | 263 (79.0%) |  |
| Symbols: ^#^ Man-Whitney-U test; *Fisher’s exact test  Abbreviations: ASA = American Society of Anaesthesiologists; BMI = Body mass index; GLIM = Global leadership initiative on malnutrition; CT = Computed tomography; SMI = Skeletal muscle index, VAT = Visceral adipose tissue; VATI = Visceral adipose tissue index; PDAC = Pancreatic ductal adenocarcinoma; AJCC = American Joint Committee on Cancer | | | | |

**SUPPLEMENTAL TABLE 3:**

**Baseline data of all patients, patients with or without GLIM malnutrition**

|  | **Total Patients (n=437)** | **With malnutrition (n=79)** | **Without malnutrition(n=358)** | **P value** |
| --- | --- | --- | --- | --- |
| **Preoperative Status** | | | | |
| Age (years) (median, LQ;UQ) | 69(61;74) | 71(63;76) | 68 (60.75;74) | .006**^#^** |
| Female (n, %) | 201 (46.0%) | 54 (68.4%) | 147 (41.1%) | .000***** |
| ASA Status (median, LQ;UQ) | 2 (2;3) | 2 (2;3) | 2 (2;3) | .212**^#^** |
| BMI (kg/m^2^) (median, LQ;UQ) | 24.24 (22.22;26.73) | 19.63 (18.61;20.76) | 24.98 (23.24;27.21) | .000**^#^** |
| Obesity (n, %) | 36 (8.2%) | 0 | 36 (10.1%) | .001***** |
| Diabetes (n, %) | 122 (27.9%) | 18 (22.8%) | 104 (29.1%) | .332***** |
| CT SMI (cm^2^/m^2^)  (median, LQ;UQ) | 44.48 (38.70;50.73) | 37.61 (34.45;41.14) | 45.78 (39.72;52.16) | .000**^#^** |
| CT VATI (cm^2^/m^2^)  (median, LQ;UQ) | 39.07 (21.20;62.07) | 13.41 (4.89;29.06) | 44.55 (27.87;68.42) | .000**^#^** |
| CT VAT (cm^2^)  (median, LQ;UQ) | 113.2(62.50;182.43) | 40.14 (13.16;82.05) | 133.6(79.14;200.98) | .000**^#^** |
| Visceral obesity (n, %) | 201 (46.0%) | 13 (16.5%) | 188 (52.5%) | .000***** |
| Neoadjuvant treatment |  |  |  |  |
| Chemotherapy (n, %) | 79 (18.1%) | 10 (12.7%) | 69 (19.3%) | .197***** |
| Radiotherapy (n, %) | 4 (0.9%) | 0 | 4 (1.1%) | 1.00***** |
| Combination (n, %) | 1 (0.2%) | 0 | 1 (0.3%) | 1.00***** |
| Preoperative biliary stenting (n, %) | 145 (33.2%) | 29 (36.7%) | 116 (32.4%) | .510***** |
| **Operation** |  |  |  |  |
| Pancreaticoduodenectomy (n, %) | 268 (61.3%) | 52 (65.8%) | 216 (60.3%) | .444***** |
| Distal Pancreatectomy (n, %) | 104 (23.8%) | 14 (17.7%) | 90 (25.1%) | .190***** |
| Total Pancreatectomy (n, %) | 65 (14.9%) | 13 (16.5%) | 52 (14.5%) | .727***** |
| Duration of Operation (min)  (median, LQ;UQ) | 379 (302;451.5) | 380 (299.75;450.25) | 376 (305;456) | .836**^#^** |
| **Histopathological data** |  | | | |
| PDAC (n, %) | 422 (96.6%) | 74 (93.7%) | 348 (97.2%) | .162***** |
| Other (n, %) | 15 (3.4%) | 5 (6.3%) | 10 (2.8%) |  |
| AJCC stage (8^th^ edition) |  | | | |
| 0 (n, %) | 3 (0.7%) | 1 (1.3%) | 2 (0.6%) | .016***** |
| IA (n, %) | 32 (7.3%) | 4 (5.1%) | 28 (7.8%) |  |
| IB (n, %) | 62 (14.2%) | 11 (13.9%) | 51 (14.2%) |  |
| IIA (n, %) | 22 (5%) | 5 (6.3%) | 17 (4.7%) |  |
| IIB (n, %) | 140 (32.0%) | 14 (17.7%) | 126 (35.2%) |  |
| III (n, %) | 144 (33.0%) | 38 (48.1%) | 106 (29.6%) |  |
| IV (n, %) | 34 (7.8%) | 6 (7.6%) | 28 (7.8%) |  |
| Perineural tumour invasion |  |  |  |  |
| Pn0 (n, %) | 87 (19.9%) | 13 (16.5%) | 74 (20.7%) | .440***** |
| Pn1 (n, %) | 350 (80.1%) | 66 (83.5%) | 284 (79.3%) |  |
| Symbols: ^#^ Man-Whitney-U test; *Fisher’s exact test  Abbreviations: ASA = American Society of Anesthesiologists; BMI = Body mass index; GLIM = Global leadership initiative on malnutrition; CT = Computed tomography; SMI = Skeletal muscle index, VAT = Visceral adipose tissue; VATI = Visceral adipose tissue index; PDAC = Pancreatic ductal adenocarcinoma; AJCC = American Joint Committee on Cancer | | | | |

**SUPPLEMENTAL TABLE 4:**

**Incidence and severity of postoperative complications in patients with or without radiographic (CT) visceral obesity**

|  | **Total patients (n=437)** | | **Visceral obesity (n=201)** | | **Without visceral obesity (n=236)** | | **P value** |
| --- | --- | --- | --- | --- | --- | --- | --- |
|  | n | % | n | % | n | % |  |
| **Surgical complications** | 296 | 67.7 | 135 | 67.2 | 161 | 68.2 | .838***** |
| *Mild (CD 1-2)* | 182 | 41.6 | 75 | 37.3 | 107 | 45.3 | .098***** |
| *Severe (CD 3-4)* | 99 | 22.7 | 50 | 24.9 | 49 | 20.8 | .359***** |
| *Death (CD 5)* | 15 | 3.4 | 10 | 5.0 | 5 | 2.1 | .119***** |
| CR-POPF (B/C) | 37 | 8.5 | 20 | 10.0 | 17 | 7.2 | .308***** |
| Postoperative biliary fistula | 12 | 2.7 | 9 | 4.5 | 3 | 1.3 | .074***** |
| Postoperative haemorrhage | 32 | 7.3 | 17 | 8.5 | 15 | 6.4 | .463***** |
| Surgical Site Infection | 55 | 12.6 | 30 | 14.9 | 25 | 10.6 | .194***** |
| *Incisional (superficial + deep)* | 23 | 5.3 | 12 | 6.0 | 11 | 4.7 | .668***** |
| *Organ/Space* | 32 | 7.3 | 18 | 9.0 | 14 | 5.9 | .270***** |
| DGE | 53 | 12.1 | 31 | 15.4 | 22 | 9.3 | .057***** |
| Reoperation | 32 | 7.3 | 19 | 9.5 | 13 | 5.5 | .141***** |
|  | | | | | | | |
| **Other complications** | 153 | 35.0 | 70 | 34.8 | 83 | 35.2 | 1.00***** |
| Diarrhoea | 57 | 13.0 | 23 | 11.4 | 34 | 14.4 | .394***** |
| Vomiting | 14 | 3.2 | 8 | 4.0 | 6 | 2.5 | .426***** |
| Renal complications | 14 | 3.2 | 11 | 5.5 | 3 | 1.3 | .015* |
| Pulmonary embolism | 7 | 1.6 | 7 | 3.5 | 0 | 0.0 | .004* |
| **Postoperative hospital stay** (median, LQ;UQ) (days) | 17 (12;23) | | 18 (13;26) | | 15(12;20) | | .000**^#^** |
| Symbols: ^#^ Man-Whitney-U test; *Fisher’s exact test  Abbreviations: CD = Clavien Dindo; CR-POPF = Clinically relevant postoperative pancreatic fistula; DGE = delayed gastric emptying | | | | | | | |

**SUPPLEMENTAL TABLE 5:**

**Incidence and severity of postoperative complications in patients with or without radiographic (CT) sarcopenic obesity**

|  | **Total patients (n=437)** | | **With sarcopenic obesity (n=104)** | | **Without sarcopenic obesity (n=333)** | | **p value** |
| --- | --- | --- | --- | --- | --- | --- | --- |
|  | n | % | n | % | n | % |  |
| **Surgical complications** | 296 | 67.7 | 71 | 68.3 | 225 | 67.6 | 1.00** |
| *Mild (CD 1-2)* | 182 | 41.6 | 36 | 34.6 | 146 | 43.8 | .111** |
| *Severe (CD 3-4)* | 99 | 22.7 | 28 | 26.9 | 71 | 21.3 | .231** |
| *Death (CD 5)* | 15 | 3.4 | 7 | 6.7 | 8 | 2.4 | .042* |
| CR-POPF (B/C) | 37 | 8.5 | 13 | 12.5 | 24 | 7.2 | .106** |
| Postoperative biliary fistula | 12 | 2.7 | 7 | 6.7 | 5 | 1.5 | .010** |
| Postoperative haemorrhage | 32 | 7.3 | 10 | 9.6 | 22 | 6.6 | .289** |
| Surgical Site Infection | 55 | 12.6 | 19 | 18.3 | 36 | 10.8 | .037* |
| *Incisional (superficial + deep)* | 23 | 5.3 | 9 | 8.7 | 14 | 4.2 | .083** |
| *Organ/Space* | 32 | 7.3 | 10 | 9.6 | 22 | 6.6 | .289** |
| DGE | 53 | 12.1 | 19 | 18.3 | 34 | 10.2 | .038** |
| Reoperation | 32 | 7.3 | 12 | 11.5 | 20 | 6.0 | .082** |
|  | | | | | | | |
| **Other complications** | 153 | 35.0 | 32 | 30.8 | 121 | 36.3 | .346** |
| Diarrhoea | 57 | 13.0 | 7 | 6.7 | 50 | 15.0 | .030** |
| Vomiting | 14 | 3.2 | 1 | 1.0 | 13 | 3.9 | .204** |
|  |  |  |  |  |  |  |  |
| **Postoperative hospital stay** (median, LQ;UQ) (days) | 17 (12;23) | | 18.5 (14;28.75) | | 16 (12;21) | | .000^#^ |
| Symbols: ^#^ Man-Whitney-U test; *Fisher’s exact test (one-sided); **Fisher’s exact test (two-sided)  Abbreviations: CD = Clavien Dindo; CR-POPF = Clinically relevant postoperative pancreatic fistula; DGE = delayed gastric emptying | | | | | | | |

**SUPPLEMENTAL TABLE 6:**

**Incidence and severity of postoperative complications in patients with or without GLIM malnutrition**

|  | **Total patients (n=437)** | | **With Malnutrition (n=79)** | | **No Malnutrition (n=358)** | | **P value** |
| --- | --- | --- | --- | --- | --- | --- | --- |
|  | n | % | n | % | n | % |  |
| **Surgical complications** | 296 | 67.7 | 58 | 73.4 | 238 | 66.5 | .287* |
| *Mild (CD 1-2)* | 182 | 41.6 | 31 | 39.2 | 151 | 42.2 | .706* |
| *Severe (CD 3-4)* | 99 | 22.7 | 23 | 29.1 | 76 | 21.1 | .139* |
| *Death (CD 5)* | 15 | 3.4 | 4 | 5.1 | 11 | 3.1 | .490* |
| CR-POPF (B/C) | 37 | 8.5 | 5 | 6.3 | 32 | 8.9 | .655* |
| Postoperative biliary fistula | 12 | 2.7 | 5 | 6.3 | 7 | 2.0 | .047* |
| Postoperative haemorrhage | 32 | 7.3 | 2 | 2.5 | 30 | 8.4 | .093* |
| Surgical Site Infection | 55 | 12.6 | 8 | 10.1 | 47 | 13.1 | .575* |
| *Incisional (superficial + deep)* | 23 | 5.3 | 3 | 3.8 | 20 | 5.6 | .780* |
| *Organ/Space SSI* | 32 | 7.3 | 5 | 6.3 | 27 | 7.5 | .816* |
| DGE | 53 | 12.1 | 8 | 10.1 | 45 | 12.6 | .703* |
| Reoperation | 32 | 7.3 | 6 | 7.6 | 26 | 7.3 | 1.00* |
|  | | | | | | | |
| **Other complications** | 153 | 35.0 | 30 | 38.0 | 123 | 34.4 | .602* |
| Diarrhoea | 57 | 13.0 | 11 | 13.9 | 46 | 12.8 | .854* |
| Vomiting | 14 | 3.2 | 0 | 0.0 | 14 | 3.9 | .084* |
|  | | | | | | | |
| **Postoperative hospital stay** (median, LQ;UQ) (days) | 17 (12;23) | | 18 (13;27) | | 16 (12;22) | | .090^#^ |
| Symbols: ^#^ Man-Whitney-U test; *Fisher’s exact test  Abbreviations: CD = Clavien Dindo; CR-POPF = Clinically relevant postoperative pancreatic fistula; DGE = delayed gastric emptying | | | | | | | |

**SUPPLEMENTAL TABLE 7:**

**Correlation of perineural tumour invasion and postoperative clinical gastrointestinal symptoms**

|  | **Total**  **(n=437)** | **Pn0**  **(n=87)** | **Pn1**  **(n=350)** | **P value** |
| --- | --- | --- | --- | --- |
| **Gastrointestinal symptoms** | | | | |
| **Vomiting** | 14 (3.2%) | 1 (1.1%) | 13 (3.7%) | .320** |
| **Diarrhoea** | 57 (13.0%) | 12 (13.8%) | 45 (12.9%) | .859** |
| **DGE** | 53 (12.1%) | 11 (12.6%) | 42 (12.0%) | .855** |
| Symbols: **Fisher’s exact test (two-sided)  Abbreviations: Pn = Perineural tumour invasion; DGE = delayed gastric emptying | | | | |

|  |  | **NI Severity Score** |  |  |
| --- | --- | --- | --- | --- |
|  |  | **n = 103** | **Median (LQ; UQ)** | **P value** |
| **Vomiting** | *Yes* | 3 | 2.30 (1.70;15.30) | .327^#^ |
|  | *No* | 100 | 8.85 (4.30;15.23) |  |
| **Diarrhoea** | *Yes* | 10 | 12.70 (1.93;20.70) | .684^#^ |
|  | *No* | 93 | 8.70 (4.15;15.15) |  |
| **DGE** | *Yes* | 6 | 6.20 (2.98;13.80) | .503^#^ |
|  | *No* | 97 | 9.00 (4.15;15.30) |  |
| Symbols: ^#^ Man-Whitney-U test  Abbreviations: NI = neural invasion | | | | |
